# Double-layered antibiotic-loaded cement spacer as a novel alternative for managing periprosthetic joint infection: an in vitro study

**DOI:** 10.1186/s13018-018-1033-5

**Published:** 2018-12-20

**Authors:** Shinsuke Ikeda, Katsufumi Uchiyama, Yojiro Minegishi, Keiko Ohno, Masaki Nakamura, Kazuhiro Yoshida, Kensuke Fukushima, Naonobu Takahira, Masashi Takaso

**Affiliations:** 1 Department of Orthopaedic Surgery, School of Medicine, Kitasato University, 1-15-1 Kitasato, Minami-ku, Sagamihara, Kanagawa 252-0374 Japan; 2 Department of Medication Use Analysis and Clinical Research, Meiji Pharmaceutical University, 2-522-1 Noshio, Kiyose-shi, Tokyo, 204-8588 Japan; 3 Department of Microbiology, School of Allied Health Sciences, Kitasato University, 1-15-1 Kitasato, Minami-ku, Sagamihara, Kanagawa 252-0373 Japan; 4 Department of Medical Engineering, School of Allied Health Sciences, Kitasato University, 1-15-1 Kitasato, Minami-ku, Sagamihara, Kanagawa 252-0373 Japan; 5 Department of Rehabilitation, School of Allied Health Sciences, Kitasato University, 1-15-1 Kitasato, Minami-ku, Sagamihara, Kanagawa 252-0373 Japan

**Keywords:** Spacer, Antibiotic, Bone cement, Periprosthetic joint infection, Vancomycin, Methicillin-resistant *Staphylococcus aureus*

## Abstract

**Background:**

Previous studies comparing antibiotic-loaded calcium phosphate cement to polymethylmethacrylate cement reported that although the former has higher elution volumes over a longer period, it is mechanically weak when used alone. To counter this problem, a double-layered antibiotic-loaded cement spacer in which calcium phosphate cement is coated with polymethylmethacrylate cement was created.

**Methods:**

In this study, we compared the double-layered spacer to the polymethylmethacrylate cement spacer in terms of eluent antibiotic concentration, bioactivity against methicillin-resistant *Staphylococcus aureus*, and mechanical strength. Double-layered and polymethylmethacrylate cement spacers that were loaded with vancomycin (VCM) were prepared and immersed in phosphate buffer for 84 days. To facilitate VCM elution from calcium phosphate cores in double-layered spacers, we also drilled multiple holes into the calcium phosphate layer from the spacer surface.

**Results:**

We found that VCM concentrations in double-layered spacer eluents were higher than those in polymethylmethacrylate cement spacer eluents. The double-layered spacer also had higher bioactivity than the polymethylmethacrylate cement spacer. Although the polymethylmethacrylate cement spacer eluent lost the ability to inhibit bacterial growth on day 56, the double-layered spacer eluent maintained this ability for the duration of our study. Finally, the double-layered spacer retained high mechanical strength throughout the study period.

**Conclusions:**

The beneficial biomechanical and drug-eluting properties of the double-layered spacer might qualify it to serve as a promising biomaterial that could be used for managing periprosthetic joint infections.

## Background

**Fig. 1:**
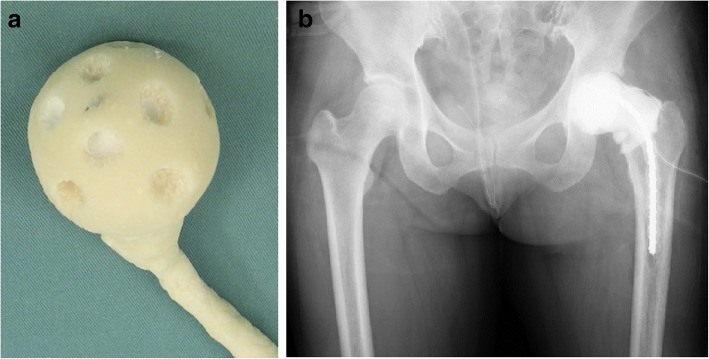
**a**, **b** D-L spacer in the clinical setting the D-L spacer comprises a calcium phosphate cement (CPC) core that is coated with PMMA and a PMMA stem. This spacer is capable of preventing discrepancies in lower limb length.

Periprosthetic joint infection (PJI) is one of the most serious postoperative complications of total joint arthroplasty, occurring in 1–2% of joint replacement surgeries [1–3]. Patients should be considered for debridement and retention of the prosthesis when PJI occurs within approximately 30 days of prosthesis implantation or fewer than 3 weeks of the onset of infectious symptoms. In contrast, patients who do not meet these criteria are usually treated using a two-stage exchange strategy that includes placement of an antibiotic-loaded cement spacer after removal of the prosthesis and thorough debridement [4]. Use of an antibiotic-loaded cement spacer is considered the standard procedure for local delivery of antibiotics. *Staphylococcus aureus* is the most common pathogen in PJI [5], with a ratio of methicillin-resistant *S. aureus* (MRSA) of approximately 50%. Despite its decreasing percentage in recent years [6], a spacer with vancomycin (VCM) (1 to 4 g per 40-g package of cement) is recommended for PJI [7]. Previous studies have reported that antibiotic-loaded calcium phosphate cement (CPC) can release a large amount of antibiotics over a long period [8, 9] and is effective for the treatment of PJI [9]. We previously used polymethylmethacrylate (PMMA)-only spacers for treatment of PJI, but because CPC was reported to have more sustained release of VCM than PMMA, we used CPC-only spacers for better treatment of PJI. However, spacers composed of only CPC are mechanically weak. We experienced breakage of the spacer during the waiting period, and we experienced difficulty removing scattered fragments of fractured CPC. Therefore, the double-layered, PMMA-coated CPC spacer (D-L spacer) was developed to achieve high-concentration and long-lasting elution of the CPC spacer with the mechanical strength of the PMMA spacer. The D-L spacer has been used in our hospital since 2008 [10]. It comprises a CPC core coated with PMMA and a PMMA stem (Fig. 1a, b). When CPC is encapsulated with PMMA, antibiotics are not released from CPC. Therefore, we attempted to drill the surface of PMMA. However, biomechanical stability decreases when holes are drilled. Fortunately, we have not experienced D-L spacer head fractures during clinical use. Therefore, we believe that this spacer might be advantageous in contrast to PMMA and CPC alone because it comprises a combination of the individual strengths of these materials. However, the strength and antibiotic elution of the D-L spacer is not known. This study aimed to compare antibiotic release, bioactivity, and mechanical strength of D-L and PMMA spacers.

## Methods

D-L spacers used in the clinical setting are not spherical, and those are with stems. Spherical spacers without stems may have different mechanical strengths compared to spacers with stems. However, the transition from the head to the stem of D-L spacer used in the clinical setting was used of only PMMA. Therefore, we considered that past data regarding strength of the transition from the head to the stem of PMMA spacers [11] can be applied to D-L spacers. In this study, we only used spherical spacer models without stems to compare the amount of VCM elution and mechanical strength of the two types of spacers.

### Preparation of the spherical femoral head spacer models

We prepared two types of femoral head spacer models: D-L spacers (five samples) and PMMA spacers (five samples). Models were prepared using CPC (Biopex; HOYA Technosurgical Corporation, Tokyo, Japan) and PMMA (Cemex RX; Exactech, Gainesville, Florida, USA) in a clean operation room. Hemispherical molds with two different diameters (40 mm and 29 mm) were also prepared using silicone resin (EXAFINE; GC Corporation, Tokyo, Japan). Then, they were sterilized and preserved.

**Fig. 2:**
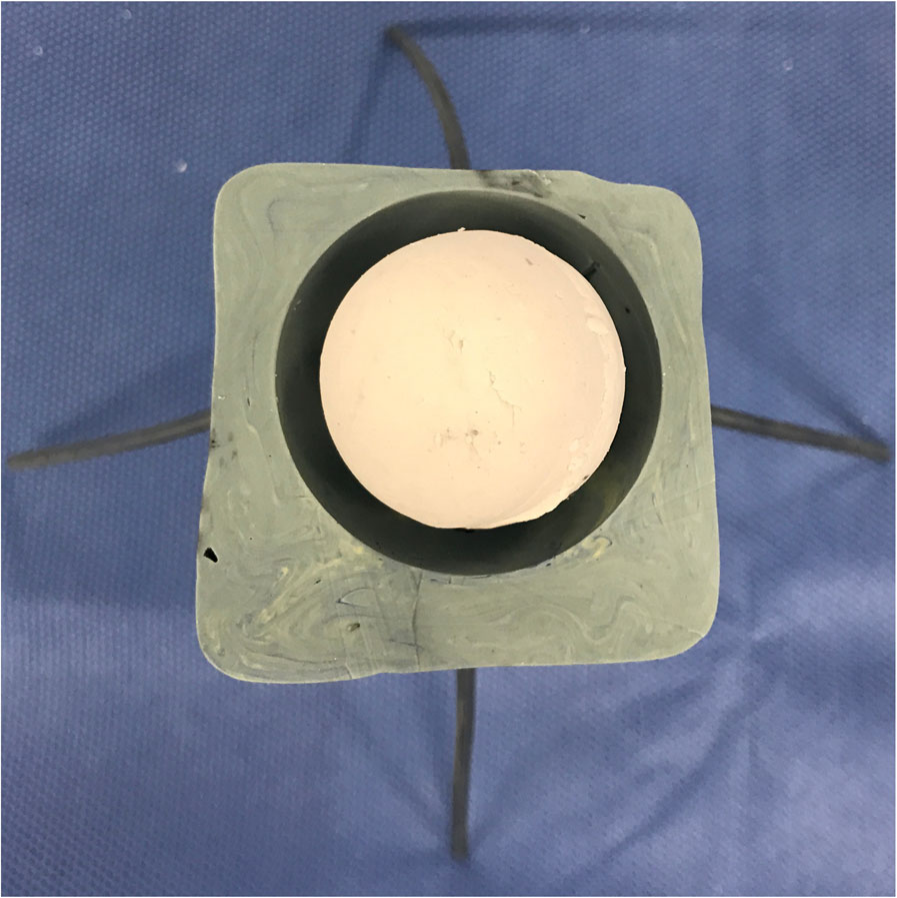
The 40-mm-diameter hemispherical mold to position the core at the center of the mold. Pins were stuck in mold, and the core was affixed to them.

To make the spherical spacer samples, we first added VCM powder (VCM 5% *w*/*w*) to both PMMA and CPC powder by manually blending. After pouring the curing solution and mixing, we manually poured CPC into a 29-mm mold and cured it to form a spherical CPC core. This was then coated with an 11-mm PMMA shell using a 40-mm mold. For the coating step, pins were stuck in the mold to ensure that the CPC core was affixed at its center during coating (Fig. 2). Next, the PMMA spacer was prepared in the same manner, although only PMMA was used during all steps. Additionally, to increase the release of antibiotics, 12 holes with a diameter of 5 mm were drilled from the surface into the core of each spacer so that the distance of the holes was consistent. We made holes in both the D-L spacer and PMMA spacer.

### VCM-containing eluent collection

Each sample was placed in sterile phosphate-buffered saline (PBS) (1.5 mL of PBS per gram of spacer) and incubated at 37 °C for 84 days; PBS was changed every 24 h. Eluent samples were taken on days 1, 3, 7, 14, 28, 56, and 84, with the same sample used to measure VCM concentrations and bioactivity at each time point. Eluents were stored at − 30 °C until analysis, which was performed in a clean environment.

### Measuring VCM concentrations using high-performance liquid chromatography

VCM concentrations in collected eluents were measured using high-performance liquid chromatography (HPLC). Briefly, the frozen eluents were milled. Then, 150 μL of each eluent and 100 μL of VCM in MeOH (50 μg/mL) as an internal standard were vortex-mixed in a micro-tube. Then, 30 μL of supernatant was injected in the HPLC system. The peak height ratios (VCM/internal standard) obtained from the standard solution were plotted against the VCM concentration to obtain a calibration curve, and the concentrations of VCM in the eluents were calculated. The HPLC conditions were as follows: mobile phase, 50 mmol/L ammonium acetate (pH 5.0)/CH_3_CN = 9/1; flow rate, 1.0 mL/min; column, TSK gel ODS–80 TM (250 mm × 4.6 mm internal diameter, 5 μm; TOSOH, Tokyo, Japan); column temperature, 40 °C; and detector, ultraviolet (246 nm).

### Evaluating bioactivity using the broth microdilution method

**Fig. 3:**
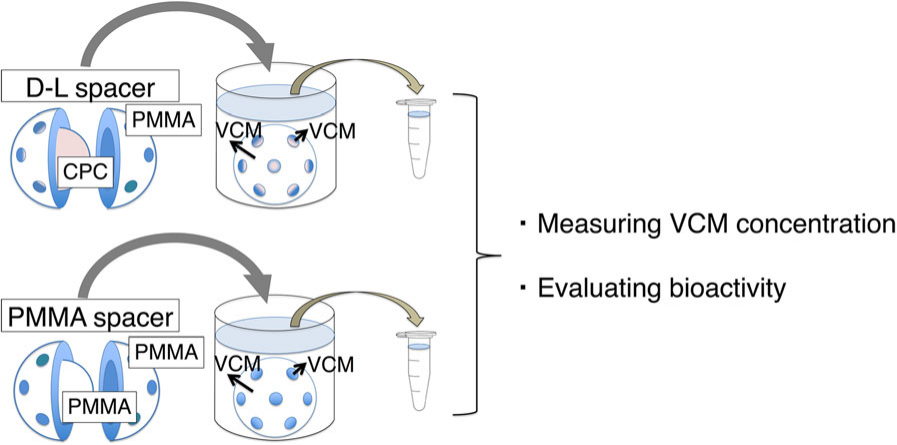
Schema demonstrating the process from preparation to measuring VCM concentrations and evaluating bioactivity

VCM bioactivity in collected eluents was evaluated using the broth microdilution method, which determines the minimum inhibitory concentration (MIC) of an antibiotic against bacteria. This method, unlike the disk diffusion method, enables the quantitative determination of antimicrobial activity. For this procedure, we used 96-well microtiter plates, cation-adjusted Mueller-Hinton broth (MHB) as the medium, and MRSA (N315 strain) that had been cultured for 24 h on an agar medium. The *S. aureus* N315 strain was isolated as a major pathogen that caused hospital-acquired infections in 1982. N315 is the first MRSA strain for which the whole genome data were published [12]. First, the bacteria were suspended in sterile physiological saline, and the solution was adjusted to an optical density of 0.26 (wavelength, 590 nm) with an absorbance meter. We then diluted 25 μL of the bacterial solution in 12 mL of MHB, with approximately 5 × 10^5^ colony-forming units/mL. VCM-containing eluent samples were subsequently serially diluted and added to the 96-well microtiter plates (10 μL per well). As a control, VCM powder was dissolved to make a fresh VCM solution. Then, it was serially diluted and added to a row of 96-well microtiter plates to determine the MIC of VCM against MRSA. Each well was also inoculated with 90 μL of the bacterial solution, making the total volume in each well 100 μL. After the plates were cultured at 37 °C for 24 h, we evaluated the bacteria growth [13]. This was performed by observing plates with the naked eye with more than 2 mm of precipitate at the bottom of wells or obvious turbidity indicating the bacteria growth. Bioactivity could be converted to the minimum concentration (μg/mL) for VCM bioactivity to perform comparisons with HPLC results by multiplying the MIC by the smallest eluent dilution rate that prevented the inhibition of MRSA growth. The schema demonstrating the experiment process from preparation to measurement of the VCM concentration and evaluation of bioactivity is shown as Fig. 3.

### Compressive strength test

After all spacers had been incubated in PBS at 37 °C for 84 days, we performed the compressive strength test. We took the samples out of PBS just before the test. Then, we performed the test immediately in the wet state. A uniaxial compressive load was applied to each spacer by the Universal Testing Machine (Instron model no. 33R 4467; Instron Corporation, Norwood, Massachusetts, USA). Spacers were positioned on the testing machine so that the drilled holes did not contact the loading plate. Compressive tests were performed with a cross-head speed of 5-mm/min, atmospheric pressure, and room temperature in the wet state. Compressive loads were applied until spacer failure occurred, and compressive strength was recorded as the maximum load applied before spacer failure.

### Statistical analysis

Eluent VCM concentrations and compressive strength were compared for D-L and PMMA spacers using the Mann-Whitney *U* test, with two-sided *P* < 0.05 regarded as statistically significant. All statistical analyses were performed using EZR (Saitama Medical Center, Jichi Medical University, Saitama, Japan), which is a graphical user interface for R (The R Foundation for Statistical Computing, Vienna, Austria). More precisely, it is a modified version of R Commander designed to add statistical functions frequently used for biostatistics [14].

## Results

### VCM concentration

**Fig. 4:**
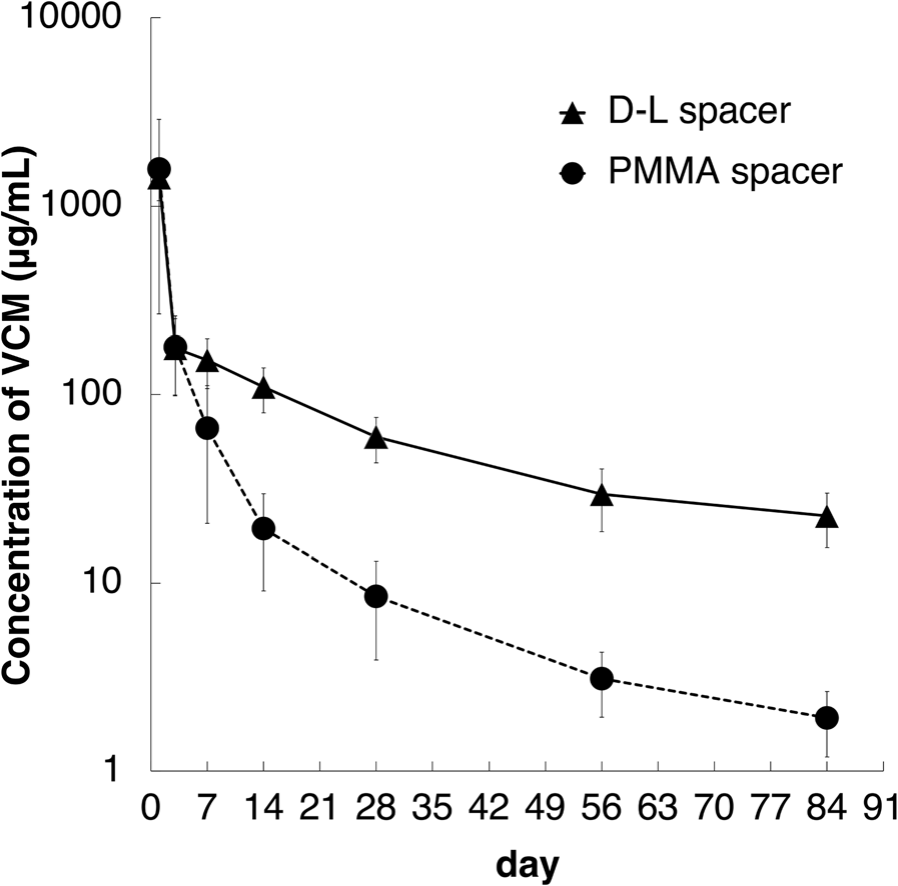
VCM release profiles (mean ± SD) in spacer eluents over an 84-day period. VCM concentrations in D-L spacer eluents (black triangle) were higher than those in PMMA spacer eluents (black circle).

VCM concentration results are shown in Fig. 4. For both D-L and PMMA spacer eluents, VCM concentrations were greatest on day 1 and decreased gradually. The VCM concentrations in D-L spacer eluents exceeded those in PMMA spacer eluents. There was a significant difference between these two VCM concentrations on and after day 7 (*P* = 0.016 on day 7; *P* = 0.0079 on and after day 14 and until day 84). This difference remained significant until day 84.

### Bioactivity

In this study, the inhibition of MRSA growth in the control row could be observed until the VCM concentration was 0.25 μg/mL. Therefore, this was determined to be the MIC of VCM against MRSA. Then, bioactivity was converted to the minimum concentration (μg/mL) for VCM bioactivity to perform comparisons with HPLC results. For example, on day 7, the D-L spacer eluent inhibited bacteria growth with up to 640 times dilution, and the PMMA spacer eluent inhibited the growth of the bacteria with up to 160 times dilution. By multiplying these with the MIC of VCM against MRSA (0.25 μg/mL), the minimum VCM bioactivity was converted to 160 μg/mL for the D-L spacers and 40 μg /mL for the PMMA spacers. Table 1 shows the crude VCM concentration and minimum VCM bioactivity values. It was demonstrated that HPLC and the broth microdilution method results were almost comparable for both D-L and PMMA spacer eluents. The bioactivity test showed that because dilution was performed only up to 1280 times, when there is more antibacterial activity, it was expressed as > 320 μg/mL. On day 56, the VCM concentration of the D-L spacer eluent was 29.4 μg/mL, and that of the PMMA spacer eluent was 3.1 μg/mL. The D-L spacer eluent had sufficient bioactivity, and that bioactivity was 20 μg/mL when converted to the VCM concentration. However, the PMMA spacer eluent on day 56 did not inhibit bacteria growth; therefore, the bioactivity was 0 μg/mL when converted to the VCM concentration. Regarding our comparison of the two spacer types, D-L spacer bioactivity significantly exceeded that of the PMMA spacer on and after day 7 and until day 84 (*P* = 0.027 on day 7; *P* = 0.012 on day 14; *P* = 0.0088 on day 28; *P* = 0.0099 on day 56; *P* = 0.0088 on day 84).

**Table 1:** VCM concentrations (mean ± SD) and minimum VCM bioactivities

	Day	1	3	7	14	28	56	84
D-L spacer (μg/mL)	VCM concentration measured by HPLC	1415.3 (± 346.9)	175.6 (± 76.2)	152.8 (± 45.2)	109.3 (± 29.3)	59.5 (± 16.1)	29.4 (± 10.8)	22.6 (± 7.3)
	Minimum VCM bioactivity	> 320	160	160	80	40	20	20
PMMA spacer (μg/mL)	VCM concentration measured by HPLC	1580.1 (± 1312.9)	179.5 (± 81.1)	66.1 (± 45.5)	19.4 (± 10.3)	8.5 (± 4.6)	3.1 (± 1.2)	1.9 (± 0.7)
	Minimum VCM bioactivity	> 320	160	40	10	2.5	0	0

Bioactivity was converted to concentration (μg/mL) by multiplying the MIC by the smallest eluent dilution rate that prevented the inhibition of MRSA growth. The values obtained by high-performance liquid chromatography (HPLC) and the broth microdilution method showed very similar values and patterns

### Compressive strength test

**Fig. 5:**
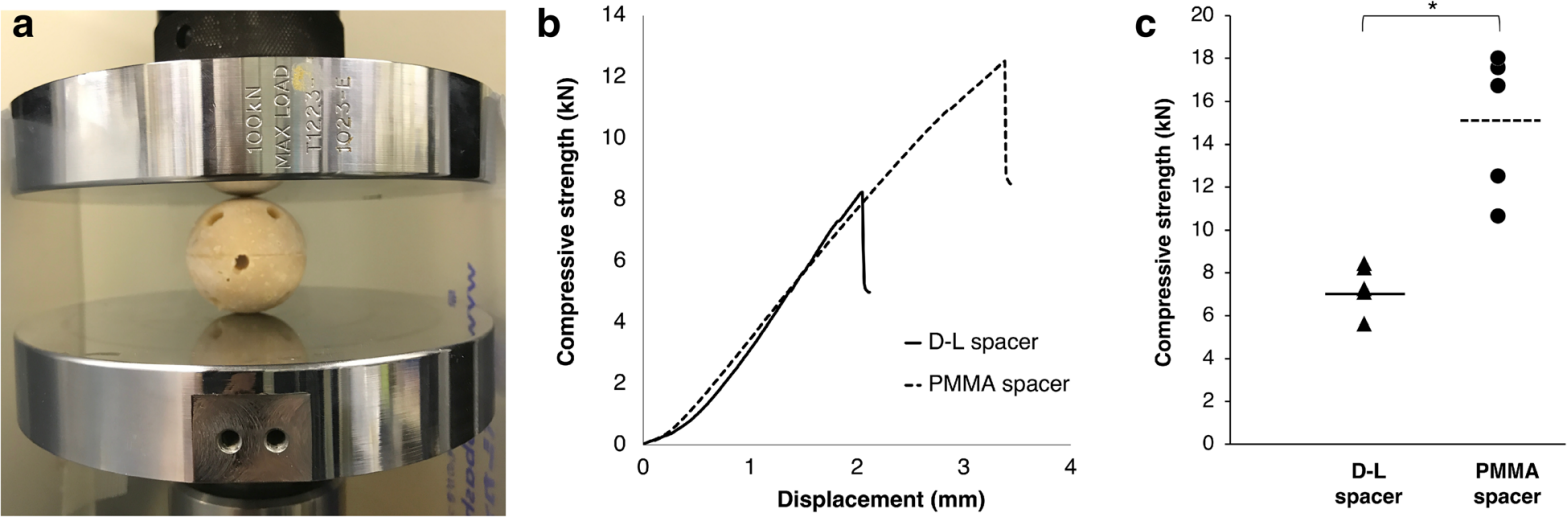
**a**–**c** Compressive strength test. **a** The sample was secured so that the holes did not contact the load surface. **b** Representative load-displacement curve of the compressive strength test. The solid line shows the load-displacement curve of D-L spacer and the dotted line shows that of PMMA spacer. These curves indicate the failure mechanisms. **c** Compressive strength of the D-L spacer (black triangle) and PMMA spacer (black circle). Dotted and solid bars show the averages of compressive strength of PMMA and D-L spacers, respectively. Compressive strength was significantly greater (*P* < 0.01) in the PMMA spacer than in the D-L spacer

Compressive strength test results are shown (Fig. 5a–c). Because we applied a load perpendicular to the spacer surfaces, cracks entered the spacers perpendicularly. We also found that the compressive strength of the D-L spacer (mean, 7.3 kN) was significantly lower than that of the PMMA spacer (mean, 15.1 kN) (*P* = 0.0079). However, it should be noted that even the weakest spacer had a compressive strength value of 5.64 kN, meaning it could withstand a load of 575 kg.

## Discussion

The D-L spacer was developed to achieve high-concentration and long-term elution of antibiotics. We made the following important observations regarding the D-L spacer in this study: (1) it released more antibiotics, (2) it was able to maintain bioactivity against MRSA, and (3) it was able to maintain high mechanical strength.

Regarding our first observation, we found that VCM concentrations of D-L spacer eluents significantly exceeded those of PMMA spacer eluents. This is consistent with the results of previous studies that showed that although PMMA allows only partial release of loaded antibiotics [15, 16], CPC allows the most loaded antibiotics to be released [8, 17, 18].

Sasaki et al. reported that the VCM elution volume from CPC on day 7 was 62.6 times that from PMMA, but that the rate thereafter decreased to 6.7 times on the day 13 [9]. However, in this study, the VCM elution volume from the D-L spacer on day 7 was 2.3 times that from the PMMA spacer, but the rate continued to increase to 11.9 times until day 84. This indicated that VCM could be released slowly and over a long period by covering CPC with PMMA. It has been reported that this antibiotic release is initially controlled to some extent by surface phenomena, whereas long-term elution depends on penetration depth, as determined by the bulk porosity of the cement used [19]. As such, we drilled multiple holes into CPC cores from the spacer surface to further facilitate VCM elution. Cement porosity, and thus the elution of antibiotics, can be increased with non-antibiotic fillers such as xylitol [20, 21]. However, fillers may degrade the mechanical properties of spacers, and the ideal amount of filler has not been established.

We also found that both D-L spacers and PMMA spacers maintained bioactivity throughout our study. Before the test, we expected that polymerization in these materials might have affected VCM activity. Specifically, because CPC does not generate the substantial amount of heat that is observed with PMMA polymerization, it does not cause the heat-induced antibiotic denaturation that may occur. It has also been reported that some organic solvents affect the stability of VCM complexes and peptide ligands [22], meaning the organic solvent contained in PMMA may have had some effect on VCM bioactivity. Considering these points, we expected to observe decreased bioactivity in the PMMA spacer. In contrast, inactivation or denaturation of loaded antibiotics, which was indicated by the presence of different peaks in HPLC analysis, was not observed. However, eluted antibiotics that show the same peak as the original loaded antibiotic do not necessarily have the same bioactivity. Therefore, we compared HPLC and bioactivity test results and found that they were comparable, and antibiotic inactivation/denaturation could be ruled out in our study.

VCM bioactivity can be affected by other factors not replicated in our study. First, biofilms that are formed by bacteria on implant surfaces can prevent the penetration of antibiotics. Nishimura et al. reported that even though antibiotics are effective against planktonic bacteria, the minimum bactericidal concentrations for biofilm bacteria of all antibiotics are high [23]. In addition, it has been reported that the protein non-binding rate of VCM in serum is only approximately 45–50% [24]. Although we could not obtain data regarding its protein-binding rate in joint fluid, the results of these studies suggest that the effect of VCM might be lost earlier in the living body than it was in our study.

It has also been reported that the MIC of VCM against MRSA is gradually increasing in several countries [25–27], which is a phenomenon known as MIC creep. We found that the minimum VCM bioactivity in PMMA barely exceeded 2 μg/mL until day 28, and it was lost by at least day 56. However, the minimum VCM bioactivity in the D-L spacer was higher than 2 μg/mL until at least day 84. If MIC creep with MRSA is considered (i.e., higher methicillin resistance levels, such as MIC of 2 μg/mL), then VCM concentrations in PMMA spacer eluents would only be effective until day 28 and are not guaranteed afterward; however, those in D-L spacer eluents would remain effective until at least day 84. This demonstrated the unreliability of the PMMA spacer and the possibility that it may be disadvantageous, especially when used for longer durations.

Finally, we found that the D-L spacer retained high mechanical strength throughout the study period, confirming our hypothesis that coating the mechanically weak CPC [28] with the stronger PMMA would result in a spacer with better mechanical properties than only CPC. This is important because the mechanical properties of cement are a primary clinical problem. Patients who are scheduled to undergo two-exchange arthroplasty for PJI are instructed to not place mechanical loads on the spacer-containing extremity while the spacer remains in the body (usually for 2 weeks to several months). However, it is possible that loads may be unconsciously placed on the affected extremity. When the patient stumbles, there could be a hip joint force of 7.2 times the body weight [29]. Although we found that maximum compressive loads were significantly lower for D-L spacers than for PMMA spacers, even the weakest spacer could withstand 5.64 kN, or a load of 575 kg. This is the value that can withstand the hip joint force when an 80-kg patient stumbles. However, even if the spherical D-L spacer can withstand an 80-kg person stumbling, there is no guarantee that the strength of the D-L spacer will be sufficient when applied clinically. Future studies are necessary.

Despite the insights provided by this study, there were some limitations. First, because we changed the solution around the spacers every day, the experimental fluid dynamics differed from actual synovial fluid dynamics in the living body. Therefore, the possibility that the dissolution kinetics in our study differed from that in a real-life environment is high. Dissolution kinetics also vary with the amount of solvent present, which we did not consider during our comparison of D-L and PMMA spacers. Furthermore, because the spacer models used in this study were smaller than spacers used clinically, it is expected that the elution amount and period are different. Second, D-L spacers that are actually used in the clinical setting are not spherical; they also have a stem. Although cement spacer fractures sometimes occur, these tend to be localized mainly in the spacer stem and neck [30], and not on the spacer head. However, in this study, we have only studied the biomechanical properties of spherical femoral head spacer models without a stem. Third, it has been reported that the amount of antibiotics, the type of cement, and the method used to create the cement are correlated with the strength of the cement [31]. Drilling several large holes can have a negative impact because these holes can act as stress concentration sites under loading. However, this was not investigated in our study. Fourth, we tested load failure once. We did not investigate the influence of repeated biomechanical loading as it happens during the clinical application. Therefore, future studies to further verify spacer strength are necessary.

## Conclusion

We compared D-L and PMMA spacer properties in this study and found that the D-L spacer was superior to the PMMA spacer in terms of elution volume and maintenance of VCM bioactivity. The D-L spacer also maintained high mechanical strength during our study duration. Therefore, we concluded that due to the beneficial biomechanical and drug-eluting properties of the D-L spacer, it might be a promising biomaterial that could potentially be used for managing PJI.

## Abbreviations

CPC: Calcium phosphate cement
D-L spacer: Double-layered spacer
HPLC: High-performance liquid chromatography
MHB: Mueller-Hinton broth
MIC: Minimum inhibitory concentration
MRSA: Methicillin-resistant *Staphylococcus aureus*
PBS: Phosphate-buffered saline
PJI: Periprosthetic joint infection
PMMA: Polymethylmethacrylate
VCM: Vancomycin
